# Dr Henry Rollin MD, FRCPsych, FRCPsych (Hon), FRCP

**DOI:** 10.1192/pb.bp.114.047423

**Published:** 2014-06

**Authors:** Robert Bluglass

**Figure:**
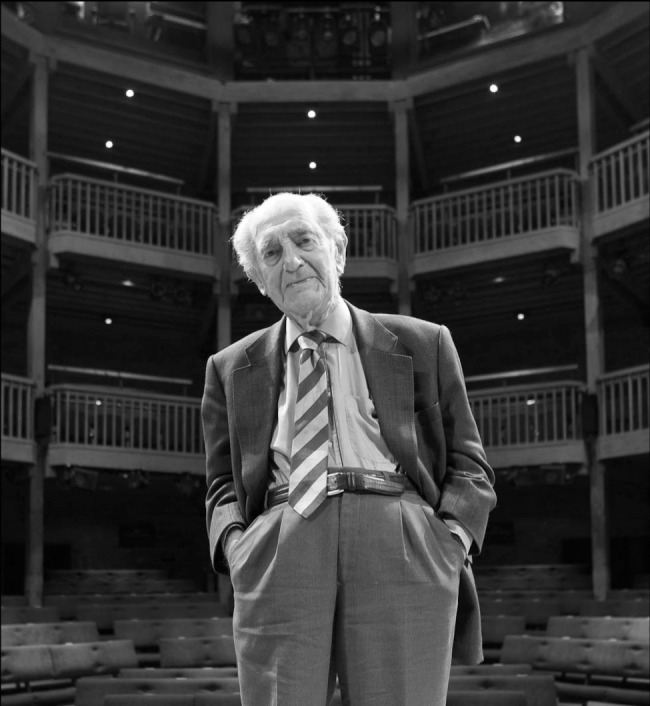
Henry Rollin at the Swan Theatre, Stratford-upon-Avon. Photo by Stewart Hemley © RSC.

Formerly Deputy Superintendent, Horton Hospital, Epsom

Henry Rollin, who died recently at the age of 102, was a medical polymath from a vanished age when psychiatrists had the time and opportunity to develop their skills by way of a wider variety of experience than is possible today. From 1948 until 1975, he was the Deputy Superintendent of Horton Hospital, Epsom, one of the largest mental hospitals in the country. Although he had himself been involved with the transformation of an old custodial asylum to a therapeutic hospital, he considered in later life that much had been lost by closing asylums and derided Enoch Powell’s wholesale destruction of them. He viewed the concept of community care with misgiving, unconvinced that the community does really care. He also deeply regretted the disasters which resulted during the era of physical methods of treatment, particularly prefrontal leucotomy, which was advocated in some cases of treatment-resistant psychotic conditions when there were few other therapeutic methods. However, he was a pioneer in the upgrading of conditions for his patients, improving their clothing, redecorating the hospital, developing new forms of occupational therapy and inviting in artists, actors and musicians. Horton became the leading hospital in the country for music therapy.

Physician superintendents at this time were requested to carry out many of the assessments of mentally abnormal offenders for the courts and Horton received many of them. This resulted in Rollin’s second career as a consultant forensic psychiatrist and for 10 years after his retirement from the NHS he became a familiar figure at Brixton Prison and in the Central Criminal Court.

Henry Rapoport Rollin was born in 1911 in Scotland, where his father, a cabinet maker from Lithuania, was a trade union leader. He was raised and educated in Leeds, where he graduated as a doctor in 1935. As a student he was lightweight boxing champion of both Leeds and the Northern universities. But his first love was literature and the arts, and he did not enjoy medical studies or the anti-Semitism that was then manifest. He had no grand plan but wanted to get to London and the theatre, music and the arts that could be enjoyed in the metropolis. He had a variety of locum jobs and then became a ship’s doctor. Although he had not considered psychiatry as a career, an opportunity presented itself in the London County Council mental health service, which resulted in the commencement of his hospital career and stimulated an enthusiasm for psychiatry. He obtained his MD for his work on Down syndrome and studied psychiatry at the Maudsley Hospital, where the acquisition of the DPM resulted in a psychiatric posting in the RAF in which he served during the war.

In 1953, he was awarded a Fulbright Fellowship to study psychosomatic medicine in the USA. He was deeply sceptical about the value of psychoanalysis, which then dominated treatment there. His interest in mentally abnormal offenders resulted in a Gwilym Gibbon research fellowship at Nuffield College, Oxford, with Professor Nigel Walker. Arising from this experience he published *The Mentally Abnormal Offender and the Law*. He always enjoyed writing and from 1961 he published more than 50 editorials in the *BMJ* and many editorials, book reviews and articles on forensic and historical topics there and in other journals. He had a vigorous literary style. Criticising government ministers for proposed cuts to the mental health services, he once wrote to the *BMJ*: ‘Doctors and their patients, may I remind them, are not packets of soap-flakes that can be moved from one shelf to the next shelf or from one shop to the next shop with impunity. Do I sound disenchanted, disillusioned, or even a trifle paranoid? I am. I bloody well am.’

Rollin was a leading figure in the fundraising necessary to elevate the Royal Medico-Psychological Association to Royal College status and in the purchase of the College’s first home at 17 Belgrave Square. He built up the College library there while serving on many committees and as Librarian for 10 years. Another of his roles was as Study Tours Secretary and he led tours to Denmark, France, Italy and Mexico. In 1976, he was elected MRCP and became a Fellow in 1993. A foundation Fellow of the Royal College of Psychiatrists, on his retirement he was elected to Honorary Fellowship, the College’s highest honour.

He served on mental health review tribunals for many years and when the Parole Board was established by Roy Jenkins he was the second psychiatric appointee and much enjoyed the opportunity to work with judges and other lawyers. He continued as a prominent psychiatric expert witness and during this time luckily survived a murderous attack by an inmate during an assessment at Brixton Prison.

Dr Rollin was a kindly and popular man who was proud of his achievements, particularly in medical journalism. The *BMJ* commissioned an autobiographical memoir *Festina Lente: A Psychiatric Odyssey* for its Memoir Series published in 1990. He was the obituaries editor of the *Psychiatric Bulletin* until shortly before his death. His 100th birthday was celebrated with an appreciation in the *British Journal of Psychiatry* by the Editor and a series of parties with his friends.

He was always an enthusiast for music, theatre and the arts and many of his friends were musicians. He relished recalling the performances of theatrical greats and was able clearly to remember a production that he had seen at the original 19th-century Shakespeare Memorial Theatre as a child. The Royal Shakespeare Company commemorated this by publishing on its website a photograph of him 2 months before his 100th birthday, standing on the site of the original stage, now in the Swan Theatre, Stratford-upon-Avon. He continued to enjoy opera and theatre in London, followed by a salt beef sandwich, until a few months ago.

Shortly before his retirement from the NHS in 1976, he had, by his own account, ‘a late flowering’ when he met Anna-Maria Tihanyi, a medical student who became a prominent consultant anaesthetist. In 1973, when he was aged 62, they married, had two children and three grandchildren, the first of whom, Leah was born on his 100th birthday. He died in his sleep on 6 February 2014. He is survived by his wife Dr Anna-Maria Rollin MBE, FRCA, children Aron and Rebecca, and three grandchildren, Leah, Ariel and Zac.

